# The required acoustic parameters simplification of invisibility cloaks and concentrators using the impedance-tunable coordinate transformation

**DOI:** 10.1038/s41598-020-79728-7

**Published:** 2021-01-13

**Authors:** Jun Cao, Fenghua Qi, Senlin Yan

**Affiliations:** 1 Department of Electronics Engineering, Nanjing Xiaozhuang University, Nanjing, 211171 People’s Republic of China; 2 Department of Physics and Institute of Theoretical Physics, Nanjing Normal University, Nanjing, 210023 People’s Republic of China

**Keywords:** Materials for devices, Theory and computation

## Abstract

Transformation acoustics, as an unconventional theory, provides a powerful tool to design various kinds of acoustic devices with excellent functionalities. However, the required ideal parameters, which are prescribed by the method, are both complex and hard to implement-even using acoustic metamaterials. Furthermore, simplified parameter materials are generally favored in transformation-acoustic design due to its easier realization with artificial structures. In this letter, we propose a coordinate transformation methodology for achieving simplified parameters by tuning the impedance distribution in the geometric limit, where the transformation media parameters can be derived by setting tunable impedance functions in the original space and a combination of suitable linear or nonlinear coordinate transformation. Based on this approach, both two-dimensional acoustic cloak and concentrators are designed with different sets of simplified parameters. Numerical simulations indicate good performance of these devices with minimized scattering at higher frequencies. The proposed method provides more opportunities to realize the designed acoustic devices experimentally, and can also be used for other transformation-acoustic designs including 3D cases.

## Introduction

The transformation-optics technique, introduced by Pendry^1^ and Leonhardt^2^, provides a unique approach to manipulate light waves. The method is based on the form-invariance of Maxwell's equations after coordinate transformation and represents a powerful approach for the design of optical devices^3–9^. Milton et al.^10^ showed that the method of coordinate transformation cannot be extended generally to suit elastic media because the elastodynamic equations have no covariant property. However, Cummer and Schurig^11^ pointed out that there is an exact analogy between 2D electromagnetics and acoustics, which was generalized to 3D acoustic-cloak design by Chen et al.^12^ Transformation acoustics has been widely used to control acoustic fields^13^. This method offers high flexibility for the guidance of acoustic waves and led to the development of acoustic metamaterials with an increased range of properties. Some examples for acoustic devices, which benefitted from this transformation technique, are the acoustic cloak^14,15^, the acoustic rotator^16^, the acoustic concentrator^17^, the acoustic lens^18,19^, and the acoustic illusion^20^.

Impedance mismatch is a common problem for practical acoustics and optical design that use the coordinate transformation method. This is especially true for the embedded coordinate-transformation method^21^, where the embedded coordinate transformation is not continuous, and the impedance of the transformation medium does not match with the surroundings in many cases^22–24^ and the resulting reflections limit possible applications. To improve the impedance match, a generalized theory of impedance-tunable transformation optics is proposed in the geometric optics limit^25^. There, the impedance function was set in the original space without changing the coordinate transformation. Impedance is inherently matched when the coordinate transformation is continuous, which is the case e.g. cloaks, concentrators, bends. However, the required ideal material parameters are complex and challenging to be realized experimentally. In addition, simplified parameters are usually preferred because of their easier practical implementation using metamaterials^3,26–29^. While impedance matching is ensured in some of these designs, impedance mismatches occur in some other cases due to limitations of the required parameter when realized with artificial structures. In this paper, for achieving impedance-matched simplified parameters in acoustic design, we propose a method of impedance-tunable transformation acoustics. According to the known coordinate transformation theory, the transformation medium in physical space is determined by placing the material in the original space and using the corresponding coordinate transformation. In Ref.^25^, It was done by adjusting the original-space setting to manipulate the impedance match without changing the coordinate transformation. Unlike Ref.^25^, in this paper we investigate impedance matching by combing the original space setting and the selected associated coordinate transformation. Based on this approach, two-dimensional acoustic cloaks and concentrators were designed. Due to the non-unique original space setting and the coordinate transformation (linear or nonlinear), a wide range of simplified parameter transformation materials can be obtained with minimized scattering, which provides more opportunities to implement the designed devices with acoustic metamaterials.

## Methods

### Theoretical design

The propagation of acoustic waves is determined by the mass density $\rho$ and the bulk modulus $\lambda$ of the involved media. The product of both determines the impedance $\eta = \sqrt {\rho \lambda }$, while the ratio of the two determines the refractive index $n = \sqrt {\frac{\lambda }{\rho }}$. To use impedance-tunable coordinate transformation, we set both the mass density $k\rho_{0}$ and bulk modulus $k\lambda_{0}$ in the original space, where the impedance coefficient $k$ is spatially dependent and a continuous function. This ensures the continuity of both original space and transformed space. For a given coordinate transformation, the mass density $\rho$ and bulk modulus $\lambda$ of the transformation medium, calculated according to Ref. 11, can be expressed as

1 $$ \begin{aligned} \frac{1}{\rho } & = \frac{{A\frac{1}{{k\rho_{0} }}A^{T} }}{\det A} \\ \frac{1}{\lambda } & = \frac{{\frac{1}{{k\lambda_{0} }}}}{\det A} \\ \end{aligned} $$

where $A$ denotes the Jacobian tensor between the original space $(x^{\prime},y^{\prime},z^{\prime})$ and the transformed space $(x,y,z)$. The transformation media are determined by both the original space setting and the associated coordinate transformation. In the coordinate-transformation theory^1^, there are no special restrictions, which transformation should be used. Even though our theory generalizes the transformation acoustics, it still lies within the framework of coordinate-transformation theory. By choosing the specific coordinate transformation and combining the appropriate impedance function setting in the original space, it is possible to obtain an impedance matched design with simplified parameters. By adjusting the impedance coefficient $k$ and selecting the corresponding coordinate-transformation, we can manipulate the impedance at the boundary and obtain several parameters of the transformation media—especially for simplified parameter materials, where the bulk modulus of the material is constant. Simplified parameter material properties are generally favored in two-dimensional transformation optical and acoustic design due to its easier implementation. Hence, it may become possible to design a near-perfect device. However, how an impedance-matched simplified parameter material can be obtained has not been discussed in detail before. In this paper, we aim to obtain a wide range of simplified parameter materials for transformation acoustic design, using impedance-tunable transformation acoustics. Furthermore, we compare the unavoidable scattering with the ideal design that uses complete parameters.

### Numerical calculation

The numerical simulation was conducted using the software COMSOL Multiphysics.

## Results

### Two-dimensional acoustic cloak

**Figure 1:**
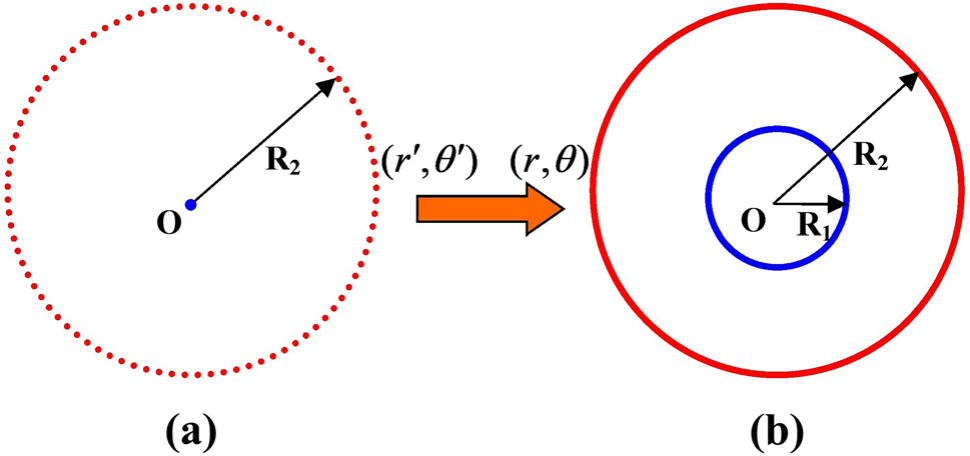
(color online) Coordinate transformation for an acoustic cloak. (**a**) original space, (**b**) transformed space.

First, we consider the acoustic cloak design. For a two-dimensional acoustic cloak, the coordinate transformation can be expressed in the region $r^{\prime} \in [0,R_{2} ]$, which is compressed into the region $r \in [R_{1} ,R_{2} ]$. Here, $r^{\prime}$ and $r$ represented the radii of the original and the transformed space, respectively. The transformation is illustrated in Fig. 1. The coordinate transformation function between original space $(r^{\prime},\theta^{\prime})$ and transformed space $(r,\theta )$ can be expressed as $r^{\prime} = f(r),\theta^{\prime} = \theta$.

According to the standard procedure used in transformation acoustics, the mass-density elements and bulk modulus, after the transformation, can be written in terms of $f(r)$ as

2 $$ \frac{1}{{\rho_{r} }} = \frac{{r^{\prime}}}{r}\frac{1}{{f^{\prime}(r)}}\frac{1}{{k\rho_{0} }},\frac{1}{{\rho_{\theta } }} = \frac{r}{{r^{\prime}}}f^{\prime}(r)\frac{1}{{k\rho_{0} }},\frac{1}{\lambda } = \frac{{r^{\prime}}}{r}f^{\prime}(r)\frac{1}{{k\lambda_{0} }} .$$

Here $f^{\prime}(r)$ is the derivative. Mathematically, there are many ways to perform a spatial transformation $r^{\prime} = f(r)$. For the linear transformation function often used before with $r^{\prime} = \frac{{R_{2} }}{{R_{2} - R_{1} }}r - \frac{{R_{1} R_{2} }}{{R_{2} - R_{1} }}$, using standard transformation acoustics (with the impedance function $k = 1$), the mass-density elements and bulk modulus can be written as:

3 $$ \frac{1}{{\rho_{r} }} = \frac{{r - R_{1} }}{r}\frac{1}{{\rho_{0} }},\frac{1}{{\rho_{\theta } }} = \frac{r}{{r - R_{1} }}\frac{1}{{\rho_{0} }},\frac{1}{\lambda } = \left( {\frac{{R_{2} }}{{R_{2} - R_{1} }}} \right)^{2} \frac{{r - R_{1} }}{r}\frac{1}{{\lambda_{0} }} $$

The above parameters for the acoustic cloak represent the ideal design case, while the impedance matches at the boundary $r = R_{2}$, where the transformation is continuous. Furthermore, the cloak is reflectionless for all frequency waves. However, both parameters are spatial functions, and controlling both the associated anisotropic mass density and the bulk modulus is difficult in experiments. To make the realization of the transformation media easier, we aim to obtain simplified parameter materials, where the bulk modulus can be constant. Then, if we let $\lambda = \lambda_{0}$, we obtain $k = \frac{{r^{\prime}}}{r}f^{\prime}(r)$. Furthermore, if we still select the linear coordinate transformation $r^{\prime} = \frac{{R_{2} }}{{R_{2} - R_{1} }}r - \frac{{R_{1} R_{2} }}{{R_{2} - R_{1} }}$, we obtain $k = (\frac{{R_{2} }}{{R_{2} - R_{1} }})^{2} \frac{{r - R_{1} }}{r}$. While $k|_{{r = R_{2} }} = \frac{{R_{2} }}{{R_{2} - R_{1} }} \ne 1$ is impedance mismatched at the boundary, this is the impedance-unmatched design case, and the resulting scattering decreases the cloaking effect. The unchanged coordinate-transformation reduces the choices for the impedance-matched transformation medium. Then, to obtain a wider, impedance-matched transformation-medium parameter, we consider a multiple-coordinate transformation, including nonlinear coordinate transformation. In this letter, we aim to obtain a simplified-parameter transformation medium, using impedance-tunable transformation acoustics, where the bulk modulus is constant and the mass density is anisotropic and spatial dependent, which is a special case of an inertial cloak as first described by Norris^30^. For this purpose, we use the impedance-tunable technique by setting the impedance function k in the original space and selecting the high-order nonlinear coordinate transformation simultaneously. To maintain the impedance matching the surrounding media at the boundary and the function $k$ at the boundary $r = R_{2}$ should be satisfied with $k = 1$. Following the implementation guideline above, we consider a quadratic transformation function $r^{\prime} = f(r) = Ar^{2} + Br + c$ for the cloak design, where $A,B,C$ are undetermined coefficients that can be determined by the parameters of the simplified transformation media. Considering to the coordinate transformation at the boundary, we can write:

4 $$ AR_{1}^{2} + BR_{1} + C = 0 $$

5 $$ AR_{2}^{2} + BR_{2} + C = R_{2} $$

We preset the bulk modulus as a constant p, and use the boundary conditions for coordinate transformation and impedance matching, then, the corresponding coordinate transformation and impedance function are obtained. Finally, the anisotropic mass density of the transformation media can be determined. Different constant *p* values correspond to different coordinate transformations, different impedance functions, and different transformation media. Here, we set $\frac{1}{\lambda } = \frac{p}{{\lambda_{0} }}$, where p is a constant. Considering the standard procedure for an impedance-tunable coordinate transformation, we get $k = \frac{{Ar^{2} + Br + C}}{r}\frac{2Ar + B}{p}$. To ensure the impedance matches at the boundary, $k|_{{r^{\prime} = R_{2} }} = 1$, we use

6 $$ 2AR_{2} + B = p .$$

Using the above three Eqs. (4), (5) and (6), we can obtain the three parameters, $A = - \frac{{R_{2} - p(R_{2} - R_{1} )}}{{(R_{2} - R_{1} )^{2} }}$,$B = p + 2R_{2} \frac{{R_{2} - p(R_{2} - R_{1} )}}{{(R_{2} - R_{1} )^{2} }}$,$C = \frac{{R_{2} - p(R_{2} - R_{1} )}}{{(R_{2} - R_{1} )^{2} }}R_{1}^{2} - pR_{1} - 2R_{1} R_{2} \frac{{R_{2} - p(R_{2} - R_{1} )}}{{(R_{2} - R_{1} )^{2} }}$.

Next, we discuss two special cases: For the first case we set $p = \frac{{R_{2} }}{{R_{2} - R_{1} }}$ and obtain $A = 0$,$B = \frac{{R_{2} }}{{R_{2} - R_{1} }}$, $C = - \frac{{R_{1} R_{2} }}{{R_{2} - R_{1} }}$, with the spatial function $r^{\prime} = f(r) = \frac{{R_{2} }}{{R_{2} - R_{1} }}r - \frac{{R_{1} R_{2} }}{{R_{2} - R_{1} }}$, which is a linear transformation. It differs from the standard transformation ($k = 1$). Here, $k = \frac{{R_{2} }}{{R_{2} - R_{1} }}\frac{{r - R_{1} }}{r}$ at the boundary $r = R_{2}$ to satisfy $k = 1$. This ensures the resulting impedance-matched simplified parameters:

7 $$ \frac{1}{{\rho_{r} }} = \frac{{R_{2} - R_{1} }}{{R_{2} }}\frac{1}{{\rho_{0} }},\frac{1}{{\rho_{\theta } }} = \frac{{R_{2} - R_{1} }}{{R_{2} }}\frac{{r^{2} }}{{(r - R_{1} )^{2} }}\frac{1}{{\rho_{0} }},\frac{1}{\lambda } = \frac{{R_{2} }}{{R_{2} - R_{1} }}\frac{1}{{\lambda_{0} }} $$

For the second case we set $p = 1$, and we obtain $A = - \frac{{R_{1} }}{{(R_{2} - R_{1} )^{2} }}$,$B = \frac{{R_{2}^{2} + R_{1}^{2} }}{{(R_{2} - R_{1} )^{2} }}$, $C = - \frac{{R_{1} R_{2}^{2} }}{{(R_{2} - R_{1} )^{2} }}$. Thus the parameters of the transformation media are

8 $$ \frac{1}{{\rho_{r} }} = \frac{1}{{(2Ar + B)^{2} }}\frac{1}{{\rho_{0} }},\frac{1}{{\rho_{\theta } }} = \frac{{r^{2} }}{{(Ar^{2} + Br + C)^{2} }}\frac{1}{{\rho_{0} }},\frac{1}{\lambda } = \frac{1}{{\lambda_{0} }}. $$

The bulk modulus of the transformation medium $\lambda$ is the same as for the background medium $\lambda_{0}$. Hence, we only need to obtain the anisotropic mass density of the metamaterials. which will make it easier to implement the designed device. The four examples for acoustic cloak design, which were discussed above, are listed in Table 1. By using different impedance functions and coordinate transformations, we can also obtain other sets of impedance-matched or unmatched cloaking materials. To ensure the transformation is monotonic for a given cloak-design with parameters $R_{1}$ and $R_{2}$, the bulk modulus, which is related to the constant p, is within a certain range. If the chosen parameter exceeds this range, we need to adjust the cloaking parameter $R_{1}$ and $R_{2}$ again.

**Table 1:** The four different cloak design.

	Coordinate transformation	Impedance function k	k value at boundary r = R_2_	Parameters of the transformation media
Case 1	$r^{\prime} = f(r) = \frac{{R_{2} }}{{R_{2} - R_{1} }}r - \frac{{R_{1} R_{2} }}{{R_{2} - R_{1} }}$	1	1	$\frac{1}{{\rho_{r} }} = \frac{{r - R_{1} }}{r}\frac{1}{{\rho_{0} }},$ $\frac{1}{{\rho_{\theta } }} = \frac{r}{{r - R_{1} }}\frac{1}{{\rho_{0} }},$ $\frac{1}{\lambda } = \left( {\frac{{R_{2} }}{{R_{2} - R_{1} }}} \right)^{2} \frac{{r - R_{1} }}{r}\frac{1}{{\lambda_{0} }}$
Case 2	$r^{\prime} = f(r) = \frac{{R_{2} }}{{R_{2} - R_{1} }}r - \frac{{R_{1} R_{2} }}{{R_{2} - R_{1} }}$	$k = (\frac{{R_{2} }}{{R_{2} - R_{1} }})^{2} \frac{{r - R_{1} }}{r}$	$\frac{{R_{2} }}{{R_{2} - R_{1} }} \ne 1$	$\frac{1}{{\rho_{r} }} = \left( {\frac{{R_{2} - R_{1} }}{{R_{2} }}} \right)^{2} \frac{1}{{\rho_{0} }},$ $\frac{1}{{\rho_{\theta } }} = \left( {\frac{{R_{2} - R_{1} }}{{R_{2} }}} \right)^{2} \left( {\frac{r}{{r - R_{1} }}} \right)^{2} \frac{1}{{\rho_{0} }},$ $\frac{1}{\lambda } = \frac{1}{{\lambda_{0} }}$
Case 3	$r^{\prime} = f(r) = \frac{{R_{2} }}{{R_{2} - R_{1} }}r - \frac{{R_{1} R_{2} }}{{R_{2} - R_{1} }}$	$k = \frac{{R_{2} }}{{R_{2} - R_{1} }}\frac{{r - R_{1} }}{r}$	1	$\frac{1}{{\rho_{r} }} = \frac{{R_{2} - R_{1} }}{{R_{2} }}\frac{1}{{\rho_{0} }},$ $\frac{1}{{\rho_{\theta } }} = \frac{{R_{2} - R_{1} }}{{R_{2} }}\left( {\frac{r}{{r - R_{1} }}} \right)^{2} \frac{1}{{\rho_{0} }},$ $\frac{1}{\lambda } = \frac{{R_{2} }}{{R_{2} - R_{1} }}\frac{1}{{\lambda_{0} }}.$
Case 4	$r^{\prime} = f(r) = Ar^{2} + Br + c$	$k = \frac{{Ar^{2} + Br + C}}{r}(2Ar + B)$	1	$\frac{1}{{\rho_{r} }} = \frac{1}{{(2Ar + B)^{2} }}\frac{1}{{\rho_{0} }},$ $\frac{1}{{\rho_{\theta } }} = \frac{{r^{2} }}{{(Ar^{2} + Br + C)^{2} }}\frac{1}{{\rho_{0} }},$ $\frac{1}{\lambda } = \frac{1}{{\lambda_{0} }}$

To test the performance of the acoustic cloak that was designed using impedance-tunable coordinate transformation, we conducted two-dimensional numerical simulations using the COMSOL Multiphysics finite-element-based electromagnetics solver. Four types of cylindrical cloaks are presented in this section. These include the ideal cloak (case 1), the impedance-unmatched linear cloak (case 2), the impedance-matched linear cloak (case 3), and the impedance-matched quadratic cloak (case 4). For the simulations, without any loss of generality, the inner and outer radii of the cloak were set to *R*_1_ = 0.1 m and *R*_2_ = 0.2 m, respectively. the computational domain was 1 m × 1 m, and was computed with approximately 240,000 elements for each case. All computational domain boundaries were perfectly matched layers.

**Figure 2:**
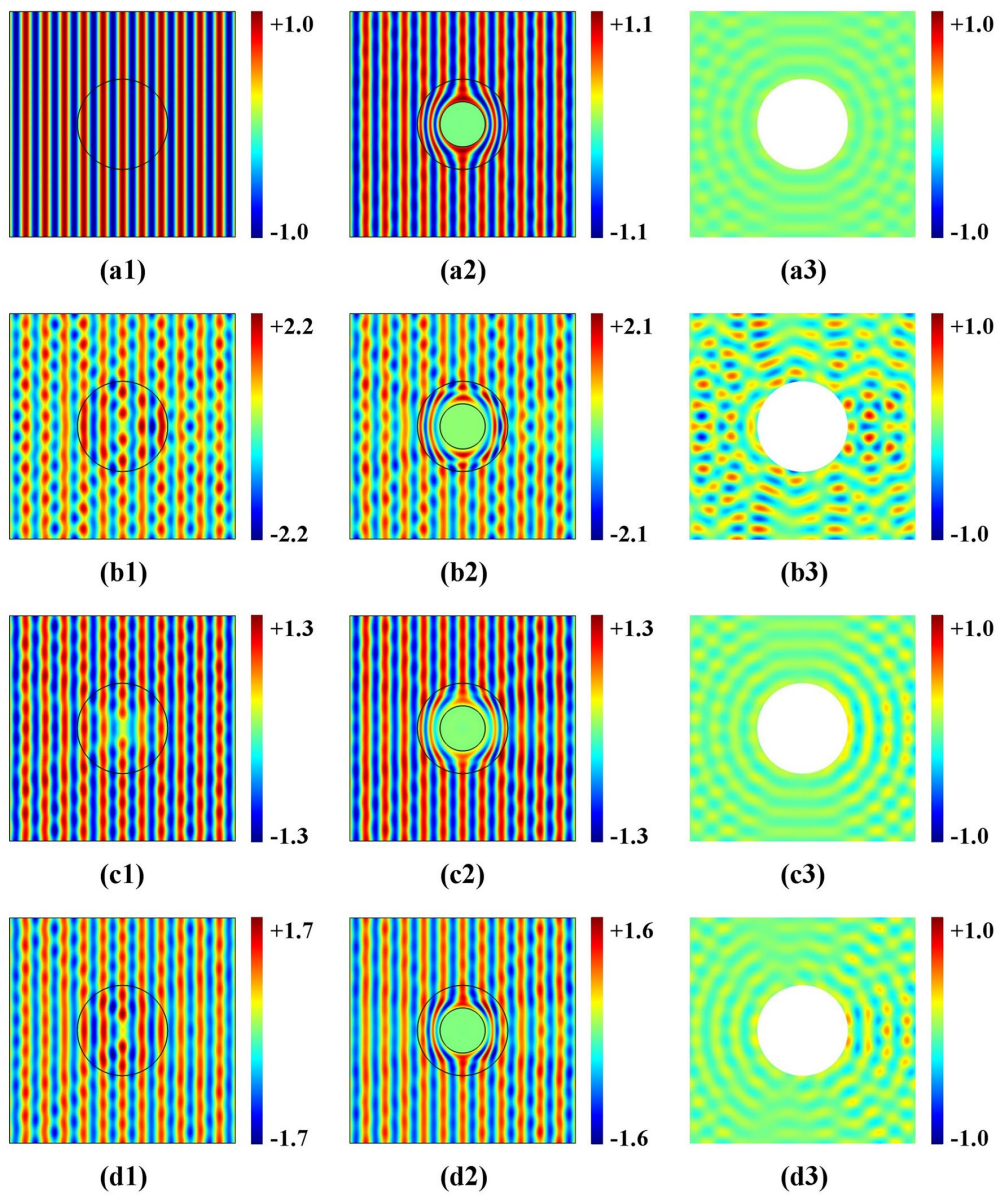
(color online) Acoustic-wave pressure distribution and scattered acoustic-wave pressure distribution using the cloak design for the four cases of different impedance function settings in the original space. (**a**) impedance function $k = 1$; (**b**) impedance function $k = (\frac{{R_{2} }}{{R_{2} - R_{1} }})^{2} \frac{{r - R_{1} }}{r}$; (**c**) impedance function $k = \frac{{R_{2} }}{{R_{2} - R_{1} }}\frac{{r - R_{1} }}{r}$; (**d**) impedance function $k = \frac{{Ar^{2} + Br + C}}{r}(2Ar + B)$.

The incident plane-wave comes from the left side with a frequency of 3 kHz. The simulated pressure-field and the corresponding scattering for four scenarios are shown in Fig. 2. The left column contains the wave simulation in the original space, while the middle-column shows the wave simulation in the physical space. The cloaked area represents an acoustic super-scatter, while the right column shows the corresponding scattered fields. Based on a comparison between the simulation results of the left and middle columns, the cloaking effect for the electromagnetic wave in physical space is consistent with propagation in the virtual space, This means that different impedance function settings determine different cloaking-effects.

For the ideal case 1, as shown in Fig. 2a1, the wave propagates through a uniform original space (k = 1) from left to right, without scattering, when transformed in physical space. Figure 2a2 illustrates that the wave is guided smoothly around the cloak, with good performance. However, a tiny distortion of the propagation-wave profile can be seen due to a computing error by the singularity for the designed material at $r = R_{1}$. Corresponding negligible scattering is shown in Fig. 2a3. For the impedance-unmatched linear cloak-design (case 2), due to the unmatched impedance setting in the original space, Fig. 2b1 reveals a destroyed propagating wave profile, which is transformed in physical space, and a reduced cloaking-performance can be seen in Fig. 2b2, while strong scattering is visible in Fig. 2b3. When the impedance is matched (case 3 and case 4), using linear and nonlinear transformation, respectively, cloaking performance is near perfect—see Fig. 2c2 and d2. Litter scattering exists because the technique uses geometric limit theory—see Fig. 2c3 and d3. Compared to the ideal case (case 1), the cloaking performance decreased slightly. However, the designed transformation media are easier to implement with a constant bulk modulus, which may enable the design of near-perfect cloaking in the future. By combing the impedance setting in the original space with suitable coordinate transformation, impedance-tuning offers more choices to obtain the impedance-matched simplified parameter materials.

**Figure 3:**
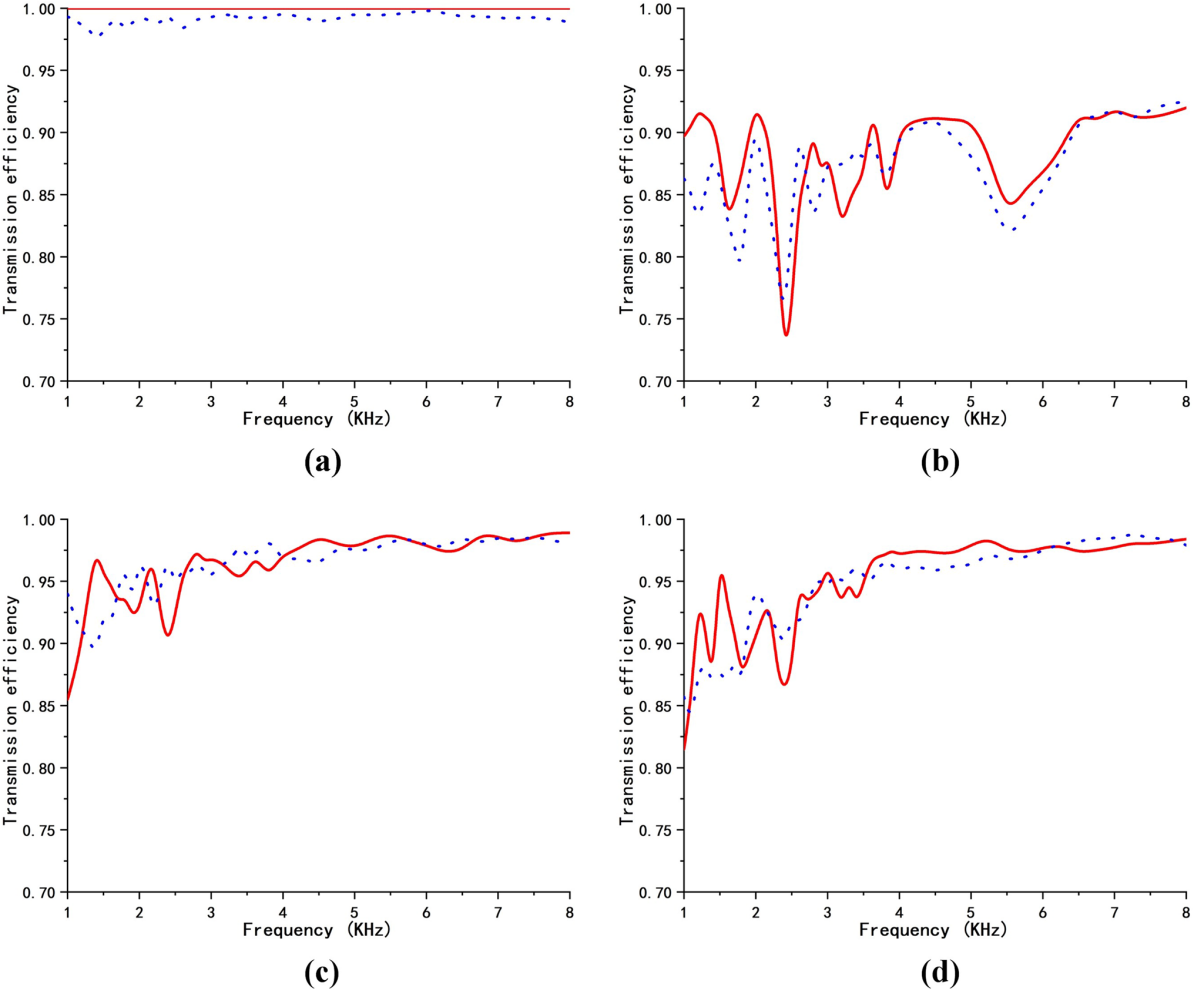
(color online) Transmission efficiency for the acoustic-wave cloak-design for the four cases. The solid red line and the dotted blue lines correspond to transmission efficiency in original space and physical space, respectively. (**a**) impedance function $k = 1$; (**b**) impedance function $k = (\frac{{R_{2} }}{{R_{2} - R_{1} }})^{2} \frac{{r - R_{1} }}{r}$; (**c**) impedance function $k = \frac{{R_{2} }}{{R_{2} - R_{1} }}\frac{{r - R_{1} }}{r}$; (**d**) impedance function $k = \frac{{Ar^{2} + Br + C}}{r}(2Ar + B)$.

Because impedance-tunable transformation acoustics is a geometric-limit theory, cloaking-performance depends on the input wave frequency. The total scattering cross section (TSCS) would be a better metric to demonstrate the efficacy of the cloak design^31,32^. In this paper, however, an alternative approximate evaluation method (by calculating the transmission efficiency) was used because of its simplicity and ease of calculation. Here, we define the transmission efficiency $\eta = \frac{{W_{2} }}{{W_{1} }}$, where $W_{1}$ is the incident wave-power at the left boundary, and $W_{2}$ is the emerging wave-power at the right side boundary. We calculate the transmission efficiency for the above four cloak-design cases in a frequency range from 1 to 8 kHz. The result is shown in Fig. 3. The solid red and the dotted blue lines correspond to the transmission efficiencies in the original and physical spaces, respectively. Theoretically, the transmission efficiency in original space and physical space should be identical. However, there are some small differences between the two efficiencies due to computation errors. For the ideal design, as shown in Fig. 3a, the transmission efficiency is 100% and independent of the frequency. For the impedance-unmatched design, as seen in Fig. 3b, the transmission efficiency changes with the wave frequency but at a lower level (around 90%), due to strong scattering. Using a tunable matched impedance, as seen in Fig. 3c and d, except for lower frequencies, the transmission efficiency becomes very high. The transmission efficiency approaches 100%, when the frequency is high enough. This means that scattering is negligible.

### Two-dimensional acoustic concentrator

**Figure 4:**
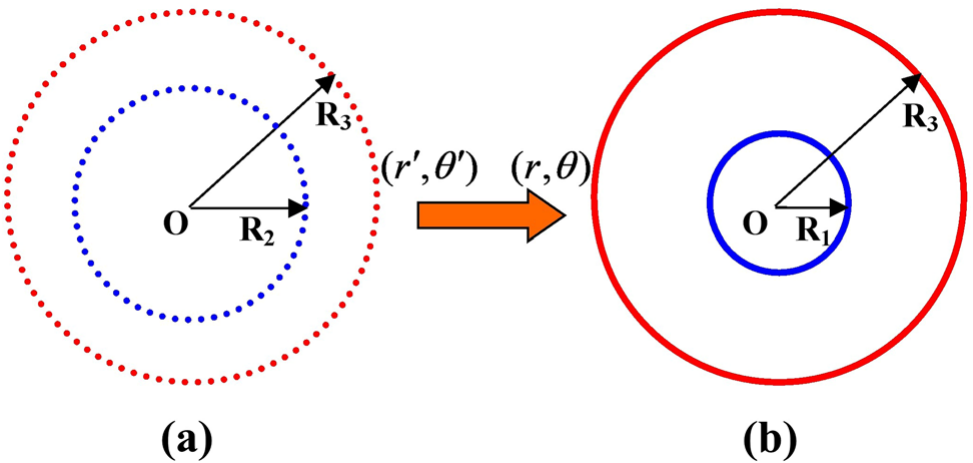
(color online) Coordinate transformation of an acoustic concentrator. (**a**) original space, (**b**) transformed space.

Beside serving as a cloak, a concentrator is also a very important devices in the acoustic design. Using the impedance-tunable technique, simplified parameters can also be obtained for concentrator designs. To design a cylindrical concentrator, the coordinate transformation can be formulated for the region $r^{\prime} \in [0,R_{2} ]$, which is compressed into the region $r \in [0,R_{1} ]$, i.e., the core region. The region $r^{\prime} \in [R_{2} ,R_{3} ]$ is focused into the region $r \in [R_{1} ,R_{3} ]$, i.e., the circular region. See Fig. 4, for a linear transformation^5^

9 $$ r^{\prime} = f(r) = \left\{ {\begin{array}{*{20}l} {\frac{{R_{2} }}{{R_{1} }}r\quad (core\;region,o < r < R_{1} )} \hfill \\ {\frac{{R_{3} - R_{2} }}{{R_{3} - R_{1} }}r + \frac{{R_{2} - R_{1} }}{{R_{3} - R_{1} }}R_{3} \quad (circular\;region,R_{1} < r < R_{3} )} \hfill \\ \end{array} } \right. .$$

For the standard transformation (k = 1), the mass-density elements, and bulk modulus of the transformation media are:

10 $$ \left\{ {\begin{array}{*{20}l} {\frac{1}{{\rho_{r} }} = \frac{1}{{\rho_{0} }},\frac{1}{{\rho_{\theta } }} = \frac{1}{{\rho_{0} }},\frac{1}{\lambda } = \frac{{R_{2}^{2} }}{{R_{1}^{2} }}\frac{1}{{\lambda_{0} }}\,\,(0 < r < R_{1} )} \hfill \\ {\frac{1}{{\rho_{r} }} = \frac{{r^{\prime}}}{r}\frac{{R_{3} - R_{1} }}{{R_{3} - R_{2} }}\frac{1}{{\rho_{0} }},\frac{1}{{\rho_{\theta } }} = \frac{r}{{r^{\prime}}}\frac{{R_{3} - R_{2} }}{{R_{3} - R_{1} }}\frac{1}{{\rho_{0} }},\frac{1}{\lambda } = \frac{{r^{\prime}}}{r}\frac{{R_{3} - R_{2} }}{{R_{3} - R_{1} }}\frac{1}{{\lambda_{0} }}\,\,(R_{1} < r < R_{3} )} \hfill \\ \end{array} } \right. $$

The concentrator is reflectionless due to the inherent impedance-matching used by the continuous coordinate-transformation itself. The parameter after transformation in the core region represents an isotropic constant and is easy to fabricate. However, in the circular region, the transformation medium has an anisotropic mass-density and spatial function. Similar to the cloak design procedure to obtain the simplified parameters, which were based on the impedance-tunable technique, by introducing the impedance function $k$ and selecting a suitable coordinate-transformation, we can obtain the constant bulk-modulus for the annular region, while the core region remains unchanged. Considering the impedance matching problem for the two boundaries $r = R_{1}$ and $r = R_{{3}}$, a high-order transformation was chosen for the concentrator design instead of quadratic function transformation. Next, we used the third-order nonlinear transformation function

11 $$ r^{\prime} = f(r) = A^{\prime}r^{3} + B^{\prime}r^{2} + C^{\prime}r + D^{\prime}\quad (R_{1} < r < R_{3} ) $$

Considering the transformation at the boundary, $$R_{2} = f(R_{1} ),$$$$R_{3} = f(R_{3} )$$, we obtain

12 $$ A^{\prime}R_{1}^{3} + B^{\prime}R_{1}^{2} + C^{\prime}R_{1} + D^{\prime} = R_{2} $$

13 $$ A^{\prime}R_{3}^{3} + B^{\prime}R_{3}^{2} + C^{\prime}R_{3} + D^{\prime} = R_{3} .$$

To derive the constant bulk-modulus $\frac{1}{\lambda } = \frac{p}{{\lambda_{0} }}$, we can formulate $k = \frac{{(A^{\prime}r^{3} + B^{\prime}r^{2} + C^{\prime}r + D^{\prime})(3A^{\prime}r^{2} + 2B^{\prime}r + C^{\prime})}}{rp}$, for the boundary $k|_{{r = R_{1} }} = 1$, and $k|_{{r = R_{3} }} = 1$. We then obtain

14 $$ 3A^{\prime}R_{1}^{2} + 2B^{\prime}R_{1} + C^{\prime} = \frac{{R_{1} }}{{R_{2} }}p $$

15 $$ 3A^{\prime}R_{3}^{2} + 2B^{\prime}R_{3} + C^{\prime} = p. $$

Using the above four Eqs. (12)-(15), we obtain $A^{\prime} = \frac{{M_{1} }}{M}$,$B^{\prime} = \frac{{M_{2} }}{M}$,$C^{\prime} = \frac{{M_{3} }}{M}$, $D^{\prime} = R_{2} - A^{\prime}R_{1}^{3} - B^{\prime}R_{1}^{2} - C^{\prime}R_{1}$, with

$$ \begin{aligned} M & = \left| {\begin{array}{*{20}c} {3R_{3}^{2} } & {2R_{3} } & 1 \\ {3R_{1}^{2} } & {2R_{1} } & 1 \\ {R_{3}^{3} - R_{1}^{3} } & {R_{3}^{2} - R_{1}^{2} } & {R_{3} - R_{1} } \\ \end{array} } \right|,\;M_{1} = \left| {\begin{array}{*{20}c} p & {2R_{3} } & 1 \\ {\frac{{R_{1} }}{{R_{2} }}p} & {2R_{1} } & 1 \\ {R_{3} - R_{2} } & {R_{3}^{2} - R_{1}^{2} } & {R_{3} - R_{1} } \\ \end{array} } \right| \\ M_{2} & = \left| {\begin{array}{*{20}c} {3R_{3}^{2} } & p & 1 \\ {3R_{1}^{2} } & {\frac{{R_{1} }}{{R_{2} }}p} & 1 \\ {R_{3}^{3} - R_{1}^{3} } & {R_{3} - R_{2} } & {R_{3} - R_{1} } \\ \end{array} } \right|,\;M_{1} = \left| {\begin{array}{*{20}c} {3R_{3}^{2} } & {2R_{3} } & p \\ {3R_{1}^{2} } & {2R_{1} } & {\frac{{R_{1} }}{{R_{2} }}p} \\ {R_{3}^{3} - R_{1}^{3} } & {R_{3}^{2} - R_{1}^{2} } & {R_{3} - R_{2} } \\ \end{array} } \right| \\ \end{aligned} $$

The solutions to the four Eqs. (12) - (15) are determined by the constant p. In other words, a different constant p corresponds to a different original space-setting and the related coordinate transformation, are resulted in a different simplified parameters material of the concentrator. As with the cloak design, for the given device parameters $R_{1} ,R_{2} ,R_{3}$, p lies within a certain range. If this range is exceeded, the above four equations cannot be solved.

**Figure 5:**
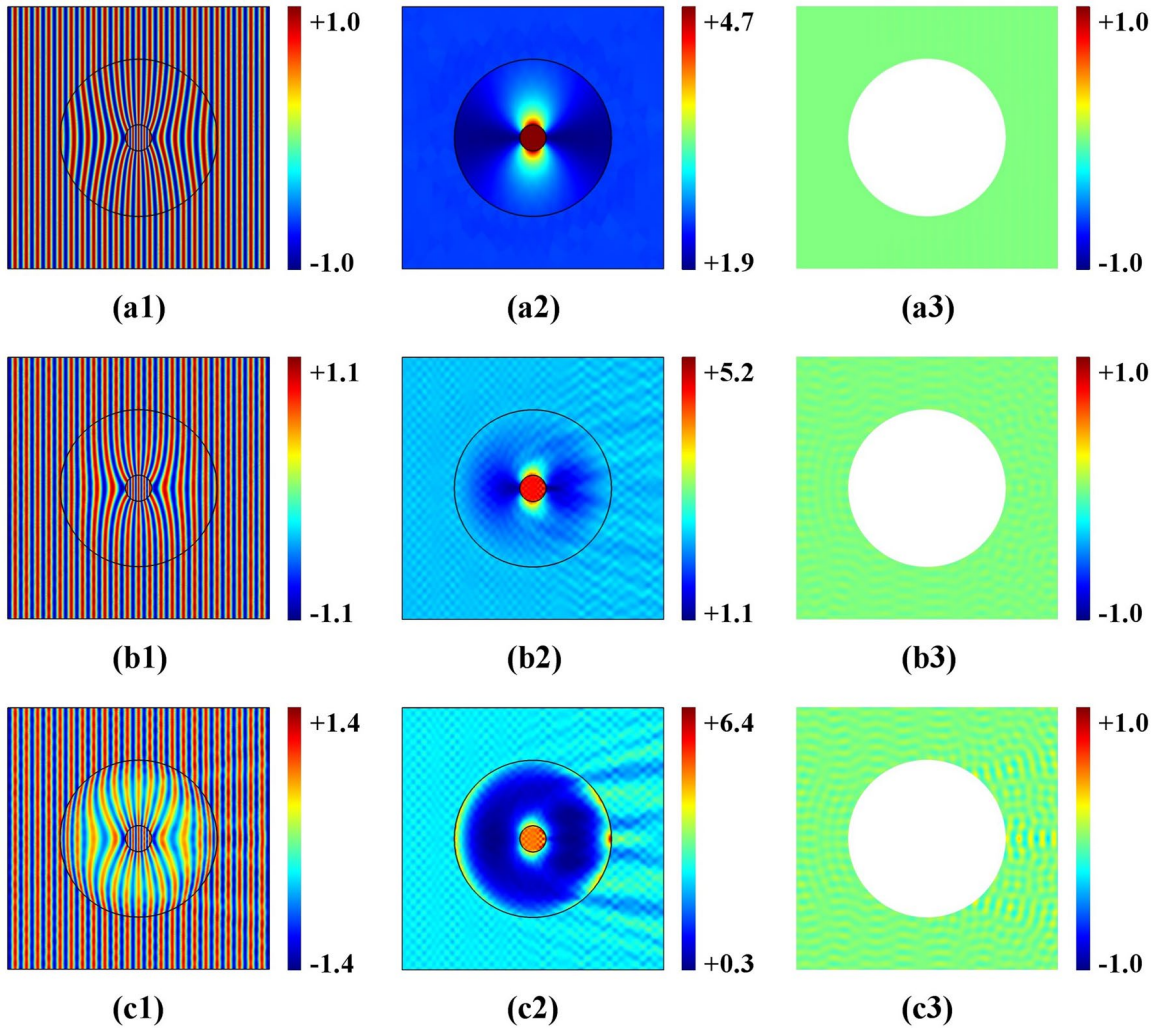
(color online) Acoustic concentrator. (**a**) ideal case, (**b**) impedance-tunable case with p = 1; (**c**) impedance-tunable case with p = 2.

In our simulations, both an ideal acoustic concentrator (k = 1) and an impedance-tunable concentrator with a different bulk modulus constant p could be simulated—without loss of generality. The radii of the concentrator were set to *R*_1_ = 0.1 m, to *R*_2_ = 0.2 m, *R*_3_ = 0.6 m, respectively. Furthermore, the computational domain was 2 m × 2 m, which was computed with approximately 310,000 elements for each case. The input wave frequency was 3 kHz. The simulated results are shown in Fig. 5. The left column shows the acoustic-wave pressure-distribution, while the middle column shows the intensity-distribution of the normalized wave, and the right column contains the scattering field distribution. For the ideal design case (k = 1), see Fig. 5a1, the acoustic plane-wave is completely focused by the concentrator into the region with a radius of $R_{1} = 0.2{\text{m}}$. Furthermore, the field intensities are substantially enhanced in the inner region with the radius $R_{1}$ for the ideal transformation material—see Fig. 5a2. Due to the uniform impedance-setting ($k = 1$) in the original space, there is no scattering—see Fig. 5a3 despite perfect concentration in the ideal design. However, both mass density and bulk modulus of the transformation material are functions of the area. However, it is difficult to realize them, using impedance-tunable coordinate transformation—see Fig. 5b and c. Simplified parameters can be used to focus waves with high efficiency, while the bulk modulus related to constant p = 1 in Fig. 5b and p = 2 in Fig. 5c. Compared to the ideal design, small scattering exists at the boundary—see Fig. 5b3 and Fig. 5c3. This is due to the geometric limit of the method.

**Figure 6:**
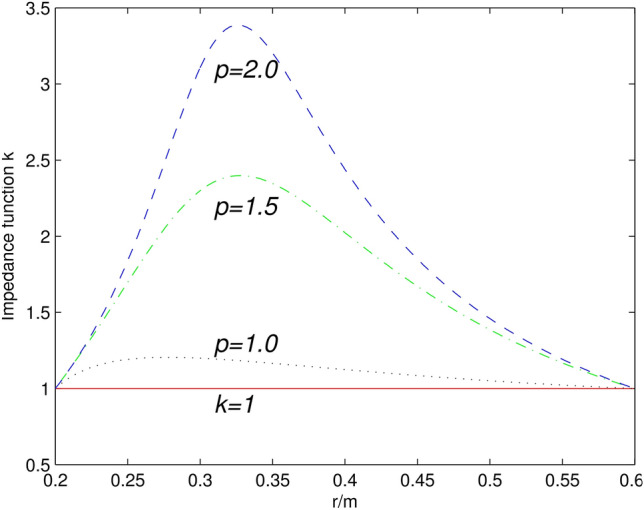
(color online) Impedance functions value distribution in the radial direction for the original space of the concentrator. The solid red line corresponds to the ideal design case with the constant k = 1. The dotted purple, dash-dotted green and dashed blue lines correspond to the impedance-tunable design case with constants p = 1, p = 1.5, and p = 2, respectively.

Clearly, scattering in the designed concentrator in Fig. 5c3 is higher than that shown in Fig. 5b3. This is because a large p causes drastic changes of the impedance within the circular area in the radial direction—see Fig. 6. To improve the declining concentration efficiency for a larger constant bulk modulus, we need to improve the frequency. When p was chosen outside the range, the equations became either insolvable or the function was not monotonic. If a larger or smaller parameter p was needed, we should change the structure values for $R_{1}$,$R_{2}$, or $R_{3}$.

## Summary

Impedance-tunable coordinate transformation technique was proposed to obtain simplified parameters for an acoustic cloak and the concentrators. Multiple impedance-matched transformation media could be obtained after adjusting the impedance setting in the original space and selecting the corresponding coordinate transformation simultaneously. Furthermore, the inherent scattering of the designed acoustic devices, due to the geometric acoustic limit, was discussed. Two-dimensional numerical simulations were performed to validate the design approach. The method can also be applied to other transformation acoustic designs, including three-dimensional cases.
